# Socioeconomic status, personality, and major mental disorders: a bidirectional Mendelian randomization study

**DOI:** 10.1038/s41537-024-00471-3

**Published:** 2024-04-27

**Authors:** Qiang Xu, Haonan Li, Dan Zhu

**Affiliations:** 1 Department of Radiology, Tianjin Key Lab of Functional Imaging & Tianjin Institute of Radiology, Tianjin Medical University General Hospital, Tianjin, China; 2 Department of Radiology, Tianjin Medical University General Hospital Airport Hospital, Tianjin, China

**Keywords:** Psychiatric disorders, Human behaviour

## Abstract

Previous research has suggested a correlation between socioeconomic status (SES) and mental diseases, while personality traits may be associated with SES and the risk of mental disorders. However, the causal nature of these associations remains largely uncertain. Our Mendelian randomization (MR) study aims to explore the bidirectional causality between SES and mental disorders, as well as to evaluate the potential mediating role of personality in these associations. Using bidirectional MR approach, we assessed the causality between SES indicators and mental disorders. We then used a two-step MR method to further investigate whether and to what extent personality mediates the causal associations in Caucasians. The forward MR analyses identified that years of education, household income, age at first birth and the Townsend deprivation index had a causal association with at least one mental disorder. The reverse MR analyses identified causal effects of genetically predicted schizophrenia, bipolar disorder, and attention deficit/hyperactivity disorder on five SES indicators. Importantly, mediation analysis showed that neuroticism partly mediated the causality of household income and years of education on major depressive disorder, respectively. In brief, our study confirmed the bidirectional relationship between SES and mental disorders. We also revealed the role of neuroticism in mediating the association between SES and major depressive disorder, highlighting the importance of considering both socioeconomic and personality factors in mental health research and interventions.

## Introduction

Socioeconomic status (SES) is an indicator of the social status or class of a group or individual, usually measured in terms of education, income, and occupation^1^. It is widely recognized that mutually reinforcing effects exist between changes in SES and the risk of mental disorders^2–5^. Personality constitutes the amalgamation of psychological traits, which is relatively stable, fundamental, and unique to an individual, potentially plays critical roles in the relationship between SES and mental disorders. Previous studies have found some evidence of associations between different SES phenotypes and personality traits (the big five personality)^6–8^. Moreover, the associations between personality and mental disorders has attracted research attention^9^, and are also well established^10,11^. However, the reliability of these studies may be affected by confounding factors and reverse causality. As a result, the causal effects and reciprocal relationship between SES and the risk of mental disorders remain largely uncertain.

Mendelian randomization (MR) is a very promising approach that attempts to measure potentially causal effects of exposures on outcomes. Single nucleotide polymorphisms (SNPs) are randomly distributed at conception and are used as instrumental variables (IVs) in MR analyses. This reduces bias in causal inference, largely independent of the effects of confounders due to other genetic variants^12^. These advantages can also be applied to mediation analysis, which demands that there be no unmeasured confounding between any of the exposure, mediator, and outcome, which is more difficult to realize in traditional observational methods^13^. MR is an effective method to investigate potential causal relationships and has been widely used in many research fields^14,15^.

**Fig. 1:**
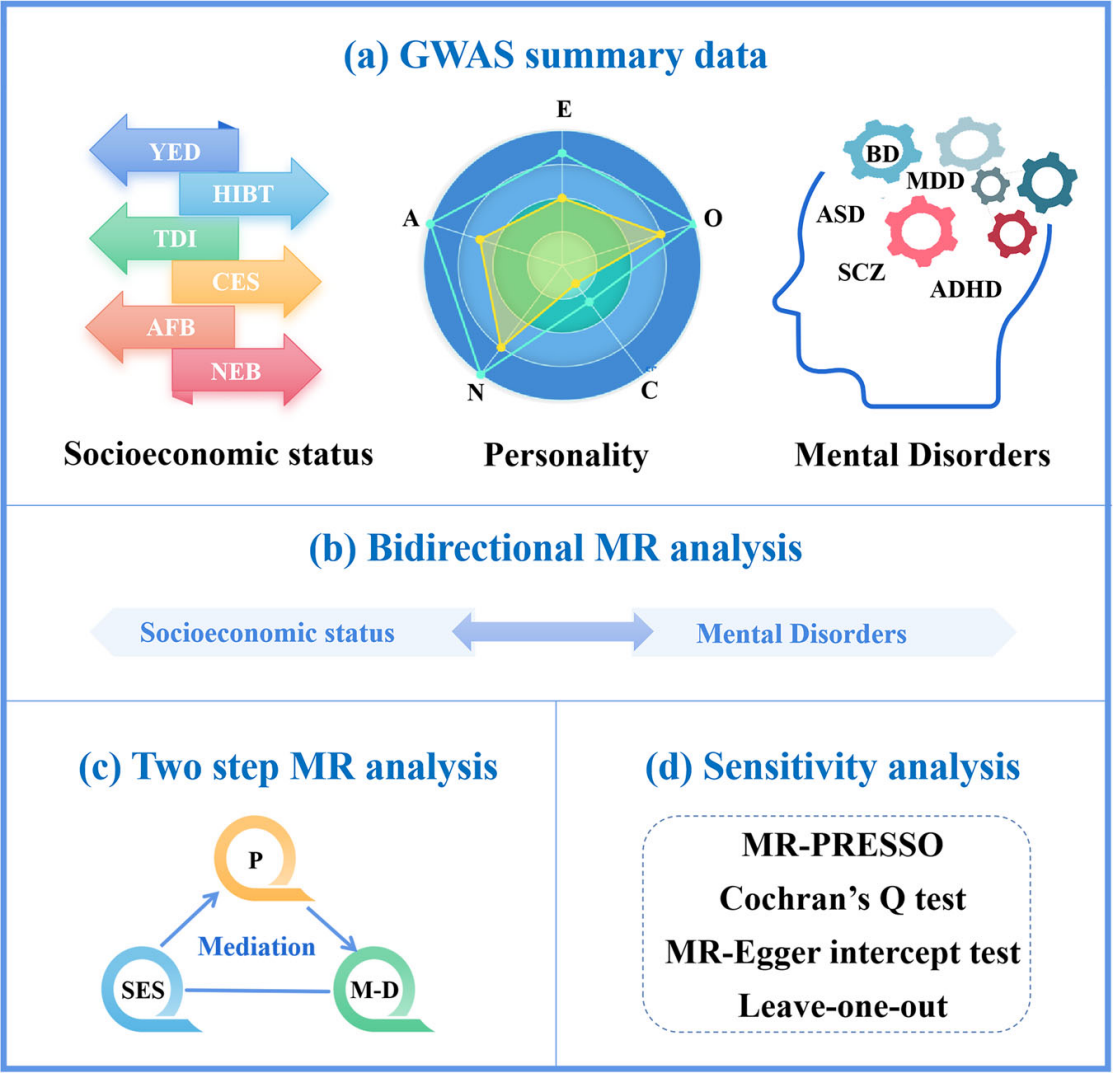
Flowchart of the MR design. **a** The GWAS summary-level data of MR study. **b** The bidirectional MR analysis between socioeconomic status and mental disorders. **c** The schematic diagram of a two-step MR and mediator analysis. **d** The sensitivity analysis methods of MR study. SES socioeconomic status, YED years of education, HIBT household income before tax, TDI Townsend deprivation index, CES current employment status, AFB age at first birth, NEB number of children ever born, P personality, A agreeableness, C conscientiousness, E extraversion, O openness, N neuroticism, M-D mental disorders, SCZ schizophrenia, BD bipolar disorder, MDD major depressive disorder, ASD autism spectrum disorder, ADHD attentiondeficit/hyperactivity disorder, MR-PRESSO Mendelian randomization-pleiotropy residual sum and outlier.

Research on the dimensional effects of SES, personality, and mental health has been limited thus far, with few studies specifically designed to explore mediation effects. In this study, our objective was to elucidate the bidirectional causality between SES and mental disorders using publicly available large-scale genome-wide association study (GWAS) data. Furthermore, we aimed to investigate the extent to which personality traits could explain the causal relationship between SES and mental disorders. Therefore, we first investigated the causal link between SES and mental disorders using a bidirectional two-sample MR method. In addition, we used a two-step MR approach to explore the mediating effect of personality in the SES-mental disorders or mental disorders-SES association. The study frame chart of MR analysis is presented in Fig. 1.

## Methods

### GWAS summary dataset for SES

GWAS provides a unique and reliable resource of summary-level data in a large sample. The following six phenotypes were selected for the genetic data of SES: Townsend deprivation index (TDI), household income before tax (HIBT), current employment status (CES), years of education (YED), age at first birth (AFB) and number of children ever born (NEB).

The genetic instrument data for the TDI, HIBT, CES and AFB were obtained from the publicly available GWAS summary data of the UK Biobank (UKB) (IEU OpenGWAS project (mrcieu.ac.uk), and the NEB were acquired from the Within Family GWAS Consortium (https://www.withinfamilyconsortium.com/). Specifically, TDI (*n* = 462,464) is a composite score based on the differences in the percentage of four variables: unemployment, overcrowded households, households without cars, and non-home ownership^16^. A higher TDI represents a higher poverty index and a lower SES^16^. HIBT (*n* = 397,751) was calculated according to the average family income before tax during the period of 2006–2010. CES (*n* = 461,242), the main types of employment can be divided into employed and non-employed. For AFB (*n* = 542,901) and NEB (*n* = 60,430), those can reflect reproductive behaviors^17^. GWAS for YED were from the Social Science Genetic Association (https://www.thessgac.org/) (*n* = 766,345), a long-term longitudinal study of people aged 30 years or older^18^.

### GWAS summary dataset for personalities

Scientific consensus has referred to the five personality traits of openness, conscientiousness, extraversion, agreeability, and neuroticism as the five-factor model^19^, also known as the Big Five personalities. The genetic instrument data for the first four personalities mentioned above were obtained from a meta-analysis of 17,375 of Caucasians, including regions in Europe, the United States, and Australia, examining the genetic variants associated with personalities^20^. The GWAS for neuroticism was obtained from a study involving twenty-one European, six American and two Australian cohorts. The total sample size was 63,661^21^. To avoid overlap between the exposure and outcome samples, all personalities GWAS summary dataset we selected is the largest GWAS dataset available except for UKB.

### GWAS summary dataset on mental disorders

Five GWAS datasets on mental disorders were all obtained from the Psychiatric Genomics Consortium (PGC). The PGC is currently the largest consortium in psychiatry, which has conducted the most influential analysis of genome-wide genomic data for mental disorders. To ensure the sample independence of exposure and outcome in the MR Analysis, we selected data from PGC-related genetic data excluding the two large cohorts of UKB and 23andMe to avoid excessive sample overlap. The final datasets therefore comprised 143,265 individuals for major depressive disorder (MDD), 77,096 individuals for schizophrenia (SCZ)^22^, 51,710 individuals for bipolar disorder (BD)^23^, 46,351 individuals for autism spectrum disorder (ASD)^24^, and 55,374 individuals for attention deficit/hyperactivity disorder (ADHD)^24^.

We restricted our analyses to data from individuals of Caucasians. All data were publicly available GWAS summary data (Supplementary Tables 1-3), and details related to ethical approval and participant consent can be found in the original GWAS publications.

### Genetic instruments selection and quality control

In each MR analysis, genetic instrumental variables (IVs) regarding exposures and outcomes were all extracted from independent GWAS summary data, without known significant data overlaps. Since the sample size of personality was not large enough, to get more SNPs, all the exposed genetic IVs were identified at least borderline significance (*p* < 5 × 10^−6^)^25^, and clumped at the threshold of linkage disequilibrium (LD) (*r*^2^ < 0.001 within 10,000 kb). If the instrumental SNPs were not available in the outcome, proxy SNPs were also searched. For the final IVs, please refer to the Supplementary Tables 4-7.

As the number of IVs increased, so did the statistical power. However, they may reduce power if small instrumental deviations are introduced^25^. Therefore, to minimize weak instrumental bias, we calculated F-statistic to assess the strength of genetic IVs in MR analysis, and the F value was above 10 is considered meaningful^26,27^. The proportion of total variation (R^2^) was calculated to indicate the proportion of variation in the exposed phenotype. The *R*^2^ and *F* were calculated using the following formula:

$${R}^{2}={\beta }^{2}\times 2\times {MAF}\times \left(1-{MAF}\right)$$

$$F=\frac{{R}^{2}\times \left(n-2\right)}{\left(1-{R}^{2}\right)}$$

where β is the effect estimate of the genetic variant, MAF is the minor allele frequency, and *n* is the sample size from exposure GWAS^26,27^.

These IVs could explain 0.15–9.08% of the variance of the exposure. The minimum F statistic for indicating the strength of these instrumental variables was 23.13, meaning that all IVs were significant for MR analysis (Supplementary Tables 8, 9).

### MR analyses

In two-sample MR analyses, the primary MR analyses approach was the inverse variance weighted (IVW) method because it provides the highest statistical power^28^. The IVW method has been widely used in many MR analyses, especially in the absence of pleiotropy^29,30^. It combines the median-based method to obtain the estimate of the causal effect and can be better at resisting pleiotropy^31^. Meanwhile, weighted median, Weighted Mode, and MR‐Egger methods were also applied to assess the causal estimates. The approach using weighted median can provide a reliable estimation of the causal effect even if up to half of the IVs are invalid^31^. The MR-Egger approach can provide a consistent causal estimate under a weak assumption^32^. Weighted mode was used as complementary analyses. Although the statistical efficacy of these methods varies, these methods can account for different pleiotropy scenarios. Additionally, we used Bonferroni correction for multiple comparisons at the *p* < 0.05/*n* (*n* = the number of exposures × the number of outcomes in MR) level of significance.

Mediation analysis was mainly implemented through two-step MR. First, based on the results of IVW method, we estimated the effect of SES and mental disorders (total effects). Then, we used multivariable mendelian randomization (MVMR) method to estimate the effect of each mediator (personality) on each outcome while correcting for instrument genetic effects on exposure^33^. For the individual mediator effect of each personality, we used the coefficient product method as the primary method for estimating indirect effects^34,35^. The ratio of indirect effects to total effects was used to estimate the proportion of the total effects mediated separately by each personality.

### Sensitivity analyses

Sensitivity analyses were implemented with Cochran’s Q test, Mendelian Randomization Pleiotropy Residual Sum and Outlier (MR-PRESSO) MR-Egger intercept test and leave-one-out analysis. Cochran’s Q test was calculated to assess the heterogeneity. The statistically significant was *p* < 0.05. If there was heterogeneity, we recalculated the results using a random effects model^30^. We reported the IVW results for the set of IVs, with outliers removed if detected, where there was evidence (MR-Egger Intercept *p* < 0.05) of horizontal pleiotropy^36^. MR-PRESSO analysis aimed to detect potentially pleiotropic outliers and recalculate the causal effect after removing the outliers^12^. In the leave-one-out sensitivity analysis, IVs were eliminated one by one, then the two-sample MR analysis was conducted based on the remaining SNPs. To assess the validity and robustness of the mediation model, we conducted the MVMR-Egger method to test whether the results of the MVMR-IVW have pleiotropy. Heterogeneity tests were also conducted based on both MVMR-IVW and MVMR-Egger methods.

All MR analysis involves performing TwoSampleMR, Mendelian Randomization, MR‐PRESSO, and MVMR R software packages. Statistical analyses were conducted by R Version 4.1.2.

## Results

### Causal effects of SES on mental disorders

**Fig. 2:**
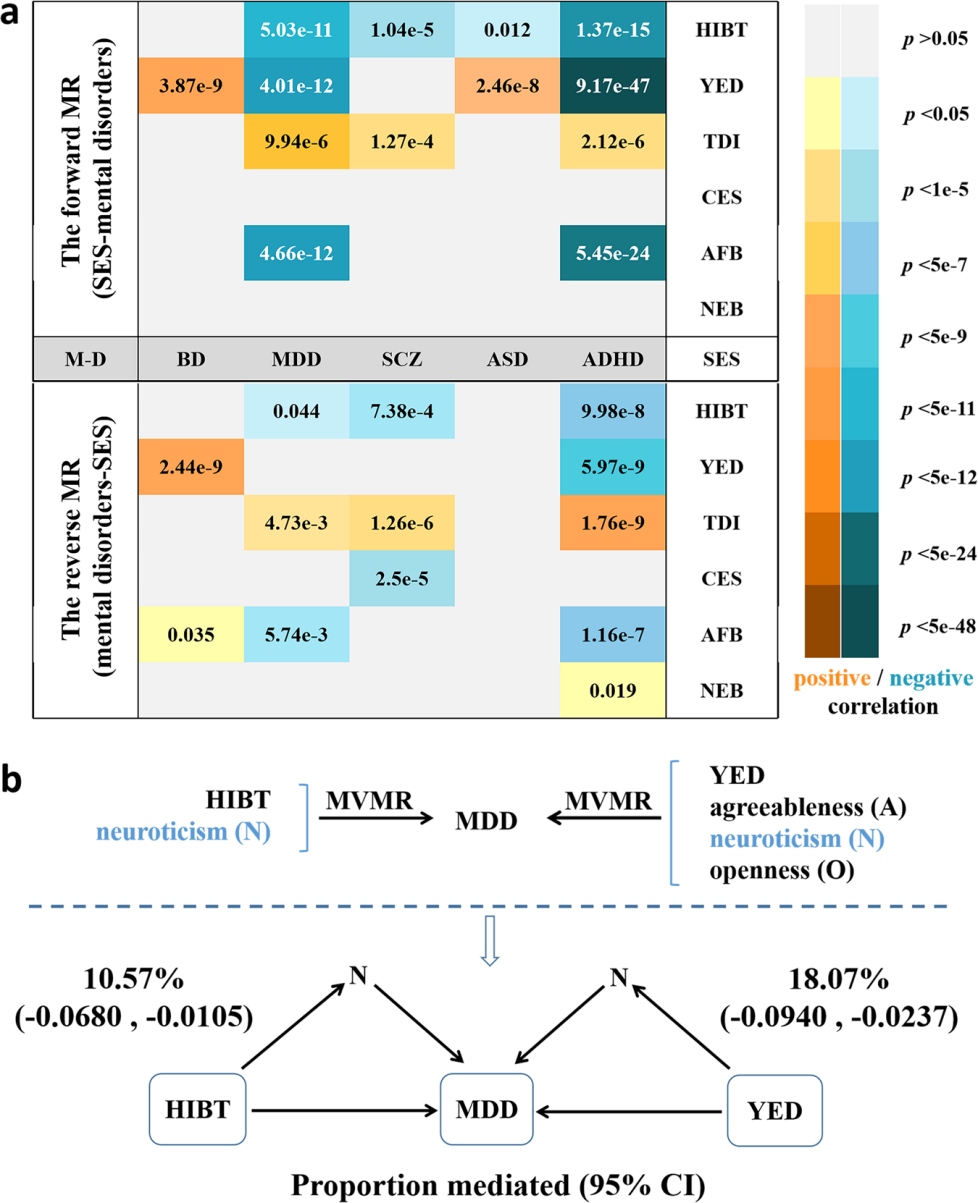
Main results of MR analysis. **a** The bidirectional MR causality (IVW method) between SES and mental disorders. **b** Mediating effect and mediating proportion of neuroticism on MDD. SES socioeconomic status, YED years of education, HIBT household income before tax, TDI Townsend deprivation index, CES current employment status, AFB age at first birth, NEB number of children ever born, M-D mental disorders, SCZ schizophrenia, BD bipolar disorder, MDD major depressive disorder, ASD autism spectrum disorder, ADHD attention deficit/hyperactivity disorder, IVW inverse variance weighted. MVMR multivariable Mendelian randomization, 95% CI 95% Confidence interval.

The causal relationships of SES on mental disorders were identified in the forward MR analyses (Fig. 2a, Supplementary Table 8). The IVW estimate and relevant sensitivity analysis suggested that the YED (OR = 0.72; *p* = 4.01 × 10^−12^), HIBT (OR = 0.69; *p* = 5.03 × 10^−11^) and AFB (OR = 0.87; *p* = 4.66 × 10^−12^) had a protective effect on MDD, while the TDI (OR = 1.66; *p* = 9.94 × 10^−6^) exerted a deleterious effect on MDD. For SCZ, HIBT (OR = 0.66; *p* = 1.04 × 10^−5^) was identified as protective factors, while TDI (OR = 1.75; *p* = 1.27 × 10^−4^) acted as a risk factor. We also showed a non-protective effect of YED on BD (OR = 1.58; *p* = 3.87 × 10^−9^) and ASD (OR = 1.53; *p* = 2.46 × 10^−8^), respectively. In addition, several characteristics of SES had strong causal effects on ADHD according to our analyses, including YED (OR = 0.31; *p* = 9.17 × 10^−47^), HIBT (OR = 0.44; *p* = 1.37 × 10^−15^), TDI (OR = 2.54; *p* = 2.12 × 10^−6^) and AFB (OR = 0.73; *p* = 5.45 × 10^−24^).

### Causal effects of SES on personality

We explored the causal relationship of partial SES phenotypes (YED, HIBT, TDI and AFB) on personality from the above results (Supplementary Table 9), as follows: Genetically predicted higher YED was associated with significantly better openness (β = 2.52; *p* = 1.77 × 10^−12^), increased agreeableness (β = 1.31; *p* = 3.42 × 10^−5^), and lower risk of neuroticism (β = -0.15; *p* = 5.33 × 10^−8^). Also, higher HIBT was associated with lower risk of neuroticism (β = -0.15; *p* = 4.06 × 10^−5^).

### Mediating effect of personality on SES-mental disorders

By using MVMR method (Supplementary Table 10), we found that neuroticism may act as potential mediators of the causal effects of TED-MDD and BIHT-MDD, respectively. Also, openness could potentially mediate the causal effect of YED-ASD. Furthermore, we validated the causal effects of neuroticism on MDD and openness on ASD employing a two-sample MR to determine the accuracy of the mediating factors. Finally, only neuroticism as a mediator was significantly associated with MDD (OR = 1.37; 95% CI: 1.13 to 1.66). Thus, by using the method of product of coefficients, neuroticism explained 10.57% (95% CI −6.80% to −1.05%) of the total effect of household income before tax and 18.07% (95% CI −9.40% to −2.37%) of years of education on MDD, respectively. (Fig. 2b, Supplementary Table 11).

### Causal effect of mental disorders on SES

The causal effects of mental disorders on SES were found in the reverse MR analyses (Fig. 2a, Supplementary Table 8). The IVW approach and relevant sensitivity analysis displayed that genetic risk of BD generally increases the YED (β = 0.03; *p* = 2.44 × 10^−9^). Higher risk of SCZ is associated with lower SES, particularly low HIBT (β = −0.01; *p* = 7.38 × 10^−4^), low CES (β = −0.01; *p* = 2.50 × 10^−5^), and a high TDI (β = 0.02; *p* = 2.50 × 10^−6^). Of course, the causality between the risk of ADHD and low SES, including short YED (β = −0.05; *p* = 5.97 × 10^−9^), low HIBT (β = −0.05; *p* = 9.98 × 10^−8^), high TDI (β = 0.04; *p* = 1.76 × 10^−9^) and early AFB (β = −0.17; *p* = 1.16 × 10^−7^), has also been proven in our analysis.

### Causal effects of mental disorders on personality

The mental disorders (exposure) selection was based solely on significant results in the MR analysis of mental disorders and SES. We only found the genetic prediction of a causal relationship of SCZ on neuroticism (β = 1.86; *p* = 9.35 × 10^−5^).

### Mediation analyses of personality on mental disorders-SES

Although the results indicated a causal relationship between SCZ and neuroticism, HIBT, CES, and TDI, respectively. We did not find any significant results for mediating effects.

### MR sensitivity analysis

Sensitivity analyses were performed to confirm the results. We performed an MR-Egger intercept test method^37^ to evaluate the mean value of the Egger intercept was non-zero, in which case the pleiotropy could be directed^38^. The MR-Egger intercept test and MR-PRESSSO were also used to check for the pleiotropy, after removing the potentially pleiotropic outliers, we recalculated the causal effect (Supplementary Tables 12, 13). By using Cochran’s Q test, despite the heterogeneity of the results between SES and different mental disorders or certain personality traits, the results did not change after adjustment using a random effects model (Supplementary Table 14). We also observed substantial heterogeneity in the pathway from SES to MDD via mediators by using MVMR method (Supplementary Table 10). However, the MR-weighted median approach was broadly consistent with the MR-IVW in terms of magnitude and direction^31^ (Supplementary Tables 8, 9), suggesting that any level of pleiotropy did not significantly bias our results^39^. Meanwhile, there was no evidence of outliers in the leave-one-out tables presented in (Supplementary 15-17). Therefore, the inferred causalities described above were plausible.

## Discussion

In our study, we initially employed a bidirectional MR method to investigate the causal relationship between SES and five major mental disorders. Furthermore, we implemented a two-step MR approach to examine the mediating role of personality in above causality. We only observed bidirectional causal relationship between YED/HIBT/TDI/AFB and ADHD, HIBT/TDI and SCZ, YED and BD. Additionally, one-way causal effects included YED/HIBT/TDI on MDD, YED on ASD, and SCZ on CES. More importantly, high neuroticism level mediated 18.07% and 10.57% of the causal effect of YED and HBIT on MDD risk, respectively.

For one thing, according to previous sociological and epidemiological studies, the protective effect of high SES is the main reason for the association between SES and mental disorders^40,41^. For example, better education and higher income have been shown to have strong protective effects^42,43^. First, our study showed that long YED could decrease the risk of MDD but increase that of BD, which is also consistent with previous studies^44,45^. The opposite effects of education on BD^46^ and MDD may be puzzling. In fact, it has been shown that BD is associated with higher intelligence prior to onset, and that excellent academic performance is associated with an increased risk of developing BD^47^. This may be because the hypomanic and depressive manifestations of BD increase access to cognitive resources, and BD patients have exaggerated emotions and extraordinary perseverance, which may have beneficial effects on learning. This seems to explain the bidirectional positive association between YED and BD risk. In contrast, the protective effect of educational attainment on MDD may reflect the benefits of its healthier lifestyles^44^. Also, parental behavior, including the level of education, can enhance development in ASD and parents play a role in many interventions^48^. Second, low HIBT levels are known to be associated with increased lifetime or incident mental disorders^49,50^, particularly MDD^49,50^ and SCZ^51^. Higher income is associated with safer places to live, healthier food and health services, as well as greater access to more advanced resources to protect against and prevent poor mental health^52^. Several studies also demonstrated that SCZ imposes a considerable economic burden, primarily due to decreased productivity resulting in reduced income. These studies were consistent with our results.

While previous studies have focused on the relationship between mental health and education or income, our study expands on this by adding additional indicators of SES such as the TDI, CES and AFB. For TDI, which is identified as a major cause of health inequality and has been linked to several mental health problems^53^. In both observational and genome-wide gene-environment interaction analyses in the UK Biobank cohort, TDI was highly correlated with psychiatric disorders^54^, including depression and BD. In our study, TDI showed a strong association with MDD but a nonsignificant association with BD, possibly because of the small samples size of GWAS for BD. In addition, studies of the TDI and SCZ are rare. Our results suggest that the TDI promotes the development of SCZ to a certain extent. Some scholars have proposed that toxic ingestion, situational crisis and psychological changes, which may stem from low SES, are risk factors for SCZ and may produce various prodromal symptoms^55^. The higher TDI, the lower SES and the higher level of poverty. The resulting series of negative changes, including psychological or emotional factors, standard of living and personal health, can promote the development of SCZ. This may be an aspect of the mutually reinforcing relationship between schizophrenia and TDI. Future studies with larger populations are necessary to further explore the underlying causes. In terms of reproductive behaviors, we have only identified a negative association of AFB with MDD, suggesting that delaying the age of delivery of the first child can mitigate the risk of mental illnesses. This was also consistent with previous observational research, and the relationship follows a monotonic pattern for males, but a parabolic pattern for females^56^. As for NEB and mental disorders, we haven’t found any connection by MR analyses. Surprisingly, no significant associations were found for CES on the five major mental disorders. However, relevant studies have shown that the risk of depression and anxiety in the unemployed are generally higher than those in the employed^57^. In the reverse analyses, we found that the risk of SCZ have a negative impact on CES, which has also been reported in related studies^58–60^. For ADHD, in line with previous findings^61^, there is a strong bidirectional associated effect between the risk of an ADHD diagnosis and SES (YED, HIBT, and TDI). We also found the causality of ADHD and AFB. Given the reproductive behavior (e.g. AFB) generally takes place during the period from adolescence to early adulthood, it tends to be associated with externalizing behaviors such as self-control, substance abuse, and psychiatric disorders (e.g. ADHD)^17^. Additionally, a population cohort study revealed that children born to parents of a younger age are at a higher risk of being diagnosed with ADHD^17^.

SES can influence the development of MDD through its impact on neuroticism, which may be supported by the following points: on the one hand, the relationship among neuroticism, SES, and MDD is intricate and interactive. A previous study has shown that individuals with low social support and high levels of neuroticism are more likely to experience MDD^62^. On the other hand, individuals with low SES often face more stressful life events and chronic stress, which can contribute to the development of neuroticism^62^. Neuroticism is associated with heightened emotional reactivity, negative cognitive biases, and maladaptive coping strategies, all of which can increase the risk of depression^63^. Finally, genetic and environmental factors, such as early life experiences and socioeconomic conditions, can shape both neuroticism and depression^64^. The interplay between these factors may further explain the link between SES, neuroticism, and depression.

This study enriches our understanding of the mediating role of neuroticism. Human beings are inherently interconnected socially, and differing socioeconomic statuses can trigger immediate psychological shifts as well as unconscious, long-term changes that impact mental health disorders to some extent. Education level and income, key indicators of SES, not only affect individuals’ fundamental survival and development but also shape their interactions within society. Education level reflects ideological and spiritual realms, while income serves as the capital and foundation for interpersonal communication. These factors directly or indirectly shape personality formation and evolution, thereby holding significant implications for our interventions in mental disorders. In summary, understanding these complex pathways is crucial for developing targeted interventions to address the mental health disparities associated with socioeconomic disadvantage.

Our study boasts several strengths. First, we explored the bidirectional causality between SES and mental disorders, and the mediating role of personality in above relationship. Second, the two step MVMR approach we used provides causal estimates, which improves the reliability of assessing the mediator’s role even in the presence of measurement errors^13^. Third, to avoid potential population heterogeneity, we limited the selection of populations to European descent. Fourth, a range of MR methods were used for validation. Sensitivity analyses were also conducted to demonstrate the stability of the results. Finally, we also extended the threshold for genetic instruments and improved the statistical power of the causal analysis.

In addition, our study has some limitations. First, to focus on the mediating effect of personality traits, the sample size of GWAS for personality traits is small, which may lead to biased results due to insufficient numbers. For instance, the GWAS of YED included 766,345 individuals, while the GWAS of personality included 17,375 individuals. This may result in unequal power for different traits or indices. Second, the participants of datasets are all of Caucasians descent, and the causality cannot be extrapolated to other races. Third, the causal conclusions of MR may potentially reflect differences in risk factors throughout the entire lifespan rather than solely at a specific point in time. Therefore, caution should be exercised when applying these findings to clinical interventions. Finally, the mediation analysis was limited to the genetic components of personality. It is important to note that mental disorders or SES are caused by a complex network of interactions among numerous factors. We should include more potentially modifiable factors to further explore the mediation effect.

In conclusion, we enriched the bidirectional causal association between SES and mental disorders while finding that SES and mental disorders also has the unique association on personality, respectively. More importantly, our study supports that interventions on neuroticism play the potential role on reducing the effect of low SES on MDD. To learn more about the underlying mechanisms, further studies are needed to assess and extend these findings.

### Supplementary information

Supplementary Tables

## Data Availability

The data analyzed in this study can be available in this published article and its supplementary information files.
